# A History of Some Cases of Diphtheritis

**Published:** 1854-04

**Authors:** Augustus M. Ayres

**Affiliations:** Salem, Miss.


### Art. III.—A HISTORY OF SOME CASES OF DIPHTHERITIS.

BY AUGUSTUS M. AYRES, M. D., OF SALEM, MISS.

The object of this communication is to give some description of the disease that heads this article, as it prevailed in this vicinity during the summer and fall of 1852. It was a visitation that will be long remembered by three families among whom its ravages were principally confined, and to none will it be more painful and bitter than to the writer of this article, a little sister having been one of its first victims.

The first cases occurred in the family of Mr. B., living four miles north-west of this place. I saw only one of the cases in his family, a negro boy about four years old, who was moribund when I first saw him. There were several cases in his family, about half of which died, which was the usual mortality. The next cases occurred in the family of Mr. S., living some four miles East of Mr. B , between whose families there had been no correspondence. The first case was an infant son of Mr. S., about twelve months old. I was called to see him in a few hours after he was taken, and learned from the family that he had had a chill followed by fever, they farther stated that they supposed his throat was sore, he having some difficulty in swallowing, on examining his throat I very readily recognized a patch of false membrane adhering to one of the tonsils (where it was invariably first deposited in all the cases I saw,) which by next examination had spread over the back part of the fauces in irregular patches. The inflammation soon extended into the posterior nares, producing a very abundant and exceedingly offensive discharge from the nose. His case progressed with but little variation, (shreds of false membrane coming away to be superceded by others,) for some four or five days, when his fever subsided with general amelioration of all the symptoms, and he had a very rapid recovery. It would be tedious to enter into a full detail of the treatment that was pursued for his case from day to day. Emetics of Ipecac were given every four or five hours, in fact at every recurrence of difficulty of breathing, two or three small mercurials were given per diem, nitrate of silver was applied in substance three times per day to the inflamed membrane, and various other local applications and gargles were used, and among the rest Bretonneau's much vaunted muriatic acid and honey.

The next case was my little sister, who was taken in precisely the same way. Some seven or eight years old. The false membrane seemed to extend much more rapidly, reaching into the nose, giving rise to the same fœtid serous discharge, and dipping, in the space of twenty-four hours, into the larynx and trachea, producing croupy symptoms of the most aggravated character, soon followed by stupor and delirium from want of proper decarbonization of the blood going to the brain, then coma with failure of the circulation and death in about sixty hours from the time of her first complaining.

The third case was a very interesting boy of Mr. S., seven or eight years of age, whose case so nearly resembled the one just reported, terminating in death in about the same time, that it would be mere repetition to enter into details..

The next cases were in the family of Mr. M., about one mile East from Mr. S., whose families were in constant correspondence. He had four cases, children, two of whom died with the same formidable array of symptoms. There were six cases in one other family; three of whom died.

I only saw the disease in two adult persons, one of whom was my much esteemed friend Dr. Davis. In his case the disease was confined to the tonsils and fauces, and showed no disposition to extend into the larynx and trachea, it lasted in his case three or four days, being so mild as not to prevent him attending his professional duties most of the time.

I saw one well marked case of diphtheritis during the past summer, in a little boy about twelve months old. The larynx and trachea soon became involved, producing the most violent croupy symptoms, terminating in death, by asphyxia, in forty-eight hours. This is the only case that has occurred within my knowledge during the past year. Most of the cases occurred in children under ten years of age.

*Treatment.*—In the mild cases very little medication was necessary, a simple mercurial cathartic with occasional cauterization of the inflamed throat with lunar caustic, was all that I found necessary. In the more malignant variety, and one of the cases I had was of this kind, all the remedies were resorted to that promised any good. I tried venesection in the second case, a stout athletic boy who had a full and bounding pulse, but before much blood had been abstracted symptoms of approaching syncope manifested themselves, and his arm was tied up; it took his system much longer than usual to recover from the syncope which made me think it at least of very doubtful propriety, and I did not resort to it in any other cases. It did not seem to exert any controlling influence whatever, as the inflammation extended with the same rapidity afterwards as it had done before. In one other case leeches were applied externally to the throat, but without producing any benefit whatever. Mercurials were given, in all the bad cases I treated, with an unsparing hand. I did not however succeed in the first case in inducing ptyalism. In the cases in which the larynx and trachea were involved they terminated in death before its influence could be brought about. Calomel seems to be the favorite remedy of all authors who have written on the disease, and from its influence in correcting the disposition in the blood to the deposit of false membrane, it occurred to me to be the remedy upon which most reliance could be placed. Most of the cases bore it well in pretty large and frequently repeated doses, but in cases terminating in croup they died before its specific influence could be induced. Emetics were resorted to in a few of the cases with a view to the detachment and expulsion of the false membrane, and frequently seemed to render good service. As to the exterior local treatment, various rubefacients, blisters, and stimulating poultices were resorted to but without effecting much good. The most important remedies, as I believe, are those which are applied directly to the inflamed parts, and of these I think nitrate of silver, either in substance or strong solution is preëminent; my usual custom was to apply it over the whole extent of the inflamed throat in view, three times per day. Powdered alum, borax, sulph.

copper, and muriatic acid diluted with honey, according to the method of BRETONNEAU, were all tried but none of them seemed to answer so well as the nitrate of silver; every application of it was followed by a considerably increased discharge from the parts to which it was applied, thus relieving the engorgement of the blood vessels and frequently promoting the detachment of large portions of false membrane to the great relief of the patient. In all the cases whenever the circulation showed a disposition to give way, stimulants such as carbonate of ammonia, brandy, quinine and the like were resorted to. Most of the cases were seen by Drs. DAVIS, WILEY and WHITTIER, none of whom had seen the disease before. I regard it when complicated with croup as the most formidable disease I have ever witnessed, not having seen one recover after the occurrence of this complication. In giving this very imperfect sketch of Diphtheritis it is with the hope of eliciting an article from some one who may be able to give a more successful mode of treatment than I, from my limited experience, have given, and if I succeed in this my highest anticipations will be realized.

